# A Meta-synthesis of nursing students’ experiences with generative artificial intelligence-assisted learning

**DOI:** 10.3389/fmed.2026.1874158

**Published:** 2026-07-16

**Authors:** Shanshan Du, Sha Wang, Fengxia Yan, Di Gong, Quan Jiang, Junnan Lin

**Affiliations:** 1 School of Nursing, Jinan University, Guangzhou, Guangdong, China; 2 Department of Paediatrics, The First Affiliated Hospital of Jinan University, Guangzhou, Guangdong, China; 3 Shenzhen Eye Hospital, Shenzhen Eye Medical Center, Southern Medical University, Shenzhen, China; 4 Department of Hepatobiliary Surgery, The First Affiliated Hospital of Jinan University, Guangzhou, Guangdong, China; 5 Department of Ophthalmology, The First Affiliated Hospital of Jinan University, Guangzhou, Guangdong, China

**Keywords:** generative artificial intelligence, meta-synthesis, nursing education, nursing students, qualitative research

## Abstract

**Objective:**

To systematically evaluate the real experiences of nursing students participating in generative artificial intelligence-assisted learning.

**Methods:**

Electronic searches were conducted in the China National Knowledge Infrastructure (CNKI), VIP Database, Scopus, Wanfang Data, and the China Biomedical Literature Database (CBM), Web of Science, PubMed, Cochrane, Embase, and CINAHL databases for qualitative studies on the experiences of nursing students with generative AI-assisted learning from the establishment of the databases to March 2026. Qualitative studies on nursing students’ experiences with generative AI-assisted learning were screened, appraised, and synthesized using thematic synthesis.

**Results:**

Eighteen studies were included in the synthesis. A total of 49 themes were identified and organized into 13 categories, leading to four integrated findings: (1) dual experience of empowerment and challenges, (2) internal conflict between technology and nursing humanism, (3) user experience differentiation amid Practical Constraints, (4) general demand for supporting systems and educational reform.

**Conclusion:**

This study found that nursing students’ experiences with generative AI are shaped by both the opportunities and challenges associated with its use in learning. These findings highlight the need for nursing educators to strengthen students’ AI literacy, critical thinking, and ethical awareness, for curriculum designers to integrate AI-related competencies into nursing curricula while maintaining a strong emphasis on humanistic care, and for policymakers to establish clear governance frameworks and educational guidelines to support the responsible use of AI in nursing education.

**Systematic review registration:**

https://www.crd.york.ac.uk/PROSPERO/view/CRD420261367837, identifier CRD420261367837.

## 1 Introduction

The rapid development of artificial intelligence (AI) has transformed many aspects of higher education, particularly teaching and learning practices (1–3). As one of the most disruptive applications in the field of AI (4), generative artificial intelligence (GenAI) is an emerging AI technology that uses large language models and related technologies to generate text, images, audio, video, and other multimodal content in response to user prompts (5–7). A recent survey reported that nearly 58% of nursing students frequently used GenAI tools, while approximately 95% accessed them through smartphones (8). Another study found that most nursing students used AI regularly, ranging from daily to monthly use (9). These findings highlight the growing influence of AI on nursing students’ learning experiences and educational practices.

In nursing education, generative artificial intelligence has been increasingly integrated into teaching and learning through a range of educational applications. Large language model (LLM)-based tools, such as ChatGPT and similar systems, are widely used to support nursing students in academic writing, concept understanding, and the development of clinical reasoning skills (10). Conversational artificial intelligence systems have also been employed to simulate patient communication scenarios, allowing students to practice nurse–patient communication, history taking, and clinical dialogue in a controlled learning environment (11). In addition, emerging multimodal AI tools support the visualization of clinical procedures and provide personalized learning feedback. By integrating textual and visual information, these tools facilitate procedural learning and enhance clinical skill acquisition (12).

Existing studies have reported mixed experiences among nursing students using generative artificial intelligence (13). While some studies highlight its potential to improve learning efficiency, support self-directed learning, and enhance access to information and feedback (14), others raise concerns regarding the accuracy of AI-generated content and the possible negative effects of overreliance on AI on critical thinking and independent problem-solving abilities (15). These contrasting findings suggest that nursing students’ experiences with generative AI are multifaceted and remain insufficiently understood.

Although qualitative studies have explored nursing students’ experiences with generative artificial intelligence, findings remain fragmented and sometimes inconsistent. Existing research has largely focused on learning outcomes, attitudes, and usage behaviors, with limited understanding of students’ broader emotional, cognitive, behavioral, and ethical experiences (16). Furthermore, variations across cultural and educational contexts make it difficult to draw comprehensive conclusions. To date, no qualitative meta-synthesis has systematically integrated this evidence. Therefore, this study aimed to synthesize existing qualitative research to provide a deeper understanding of nursing students’ experiences with generative AI.

Meta-synthesis provides a rigorous approach for integrating findings from multiple qualitative studies and generating higher-order interpretations that extend beyond individual study contexts. Given the rapid development and complexity of generative artificial intelligence in nursing education, this approach is particularly well suited to synthesizing fragmented evidence and developing a comprehensive understanding of students’ experiences. Therefore, this study aimed to synthesize qualitative evidence to gain a deeper understanding of nursing students’ experiences with generative AI in learning.

## 2 Materials and methods

### 2.1 Inclusion and exclusion criteria

The inclusion and exclusion criteria were developed using the SPIDER framework (Sample, Phenomenon of Interest, Design, Evaluation, and Research type), which is widely used to guide qualitative and mixed-methods evidence synthesis (17). The sample (S) consisted of nursing students enrolled in nursing education programs. The phenomenon of interest (PI) focused on nursing students’ perceptions, attitudes, and experiences related to generative AI–assisted learning in academic settings. The design (D) included qualitative studies and the qualitative components of mixed-methods studies, such as phenomenology, grounded theory, descriptive qualitative research, case studies, and action research. The evaluation (E) encompassed students’ experiences, perceptions, attitudes, and responses to the use of generative AI in learning. The research type (R) was restricted to qualitative research and qualitative data derived from mixed-methods studies. Studies were excluded if they were duplicates, had incomplete reporting, lacked full-text availability, were published in languages other than Chinese or English, included mixed-methods designs without extractable qualitative data, or were rated as low methodological quality (Grade C). This review was prospectively registered with the International Prospective Register of Systematic Reviews (PROSPERO; Registration No. CRD420261367837).

### 2.2 Search strategy

Computer-based searches were conducted in the following databases: CNKI, VIP, Scopus, Wanfang, Chinese Biomedical Literature, Web of Science, PubMed, Cochrane, Embase, and CINAHL, to collect qualitative studies on nursing students’ experiences with generative AI-assisted learning from the establishment of each database to March 2026. A search strategy combining subject terms and free words was formulated. In addition, snowball sampling was conducted. The reference lists of included studies and relevant reviews were manually searched to identify potentially missed studies. Including the following keywords:

“Nurse,” “nursing,” “Nurs*,” “Nursing major,” “Nursing Students,” “nursing student*,” “Nursing Education,” “Artificial Intelligence,” “machine intelligence,” “artificial intelligence,” “AI,” “generative AI,” “machine learning,” “large language models,” “Generative,” “education,” “teaching,” “Educational experience,” “student experience,” “qualitative research,” “qualitative study,” “interview*,” “focus group*,” “phenomenology,” “grounded theory,” “thematic analysis,” “experience*,” “feel*,” “attitude*,” “view*.” For example, the search strategy used in PubMed is provided in Supplementary Material 1.

### 2.3 Literature screening and data extraction

This review was conducted and reported in accordance with the PRISMA 2020 (Preferred Reporting Items for Systematic Reviews and Meta-Analyses 2020) guidelines (18). Two researchers, both trained in evidence-based methodology, independently screened the literature according to the predefined inclusion and exclusion criteria and extracted the relevant data. Titles and abstracts were screened first, followed by full-text review. Any disagreements were resolved through discussion or consultation with a third reviewer. To enhance reflexivity, all researchers had prior experience with generative AI–assisted teaching and had conducted related research. The extracted data included the author, publication year, country, research methodology, data collection methods, participants, phenomena of interest, and main findings. This review was conducted and reported in accordance with the Enhancing Transparency in Reporting the Synthesis of Qualitative Research (ENTREQ) statement (Tong et al.) (19). The completed ENTREQ checklist is available in Supplementary Material 2.

### 2.4 Literature quality evaluation method

Two researchers independently assessed the methodological quality of the included studies using the Joanna Briggs Institute (JBI) Critical Appraisal Checklist for Qualitative Research (2016). Any disagreements were resolved through discussion with a third reviewer until consensus was reached. The checklist comprises 10 items, each rated as “Yes,” “No,” or “Unclear.” Studies were graded as A when all criteria were met, B when some criteria were met, and C when none of the criteria were met.

### 2.5 Meta-synthesis method

This study employed a thematic analysis approach to synthesize the qualitative data from the included studies (20). Two researchers, both trained in qualitative research methodology and experienced in reading English academic literature, independently translated, read, and interpreted the included studies. The findings of each study were extracted and interpreted separately. Any ambiguities in translation or interpretation were discussed and resolved through consensus, and consultation with an academic English specialist was sought when necessary. In addition, the reference lists of all included studies and relevant reviews were manually searched to identify potentially relevant studies. Findings with similar meanings were then grouped into preliminary categories. Repeated coding and comparison were undertaken throughout the synthesis process. As additional studies were analyzed, no substantially new themes emerged, indicating that an adequate level of conceptual saturation had been achieved. Any discrepancies were resolved through consensus discussions, and when necessary, by consulting a third reviewer. The discrepancy resolution process involved detailed discussions to reach agreement, ensuring the objectivity of inclusion decisions. Based on this, higher-level comprehensive results were formed by analyzing the logical relationships between the categories.

## 3 Results

### 3.1 Literature search and screening results

**FIGURE 1:**
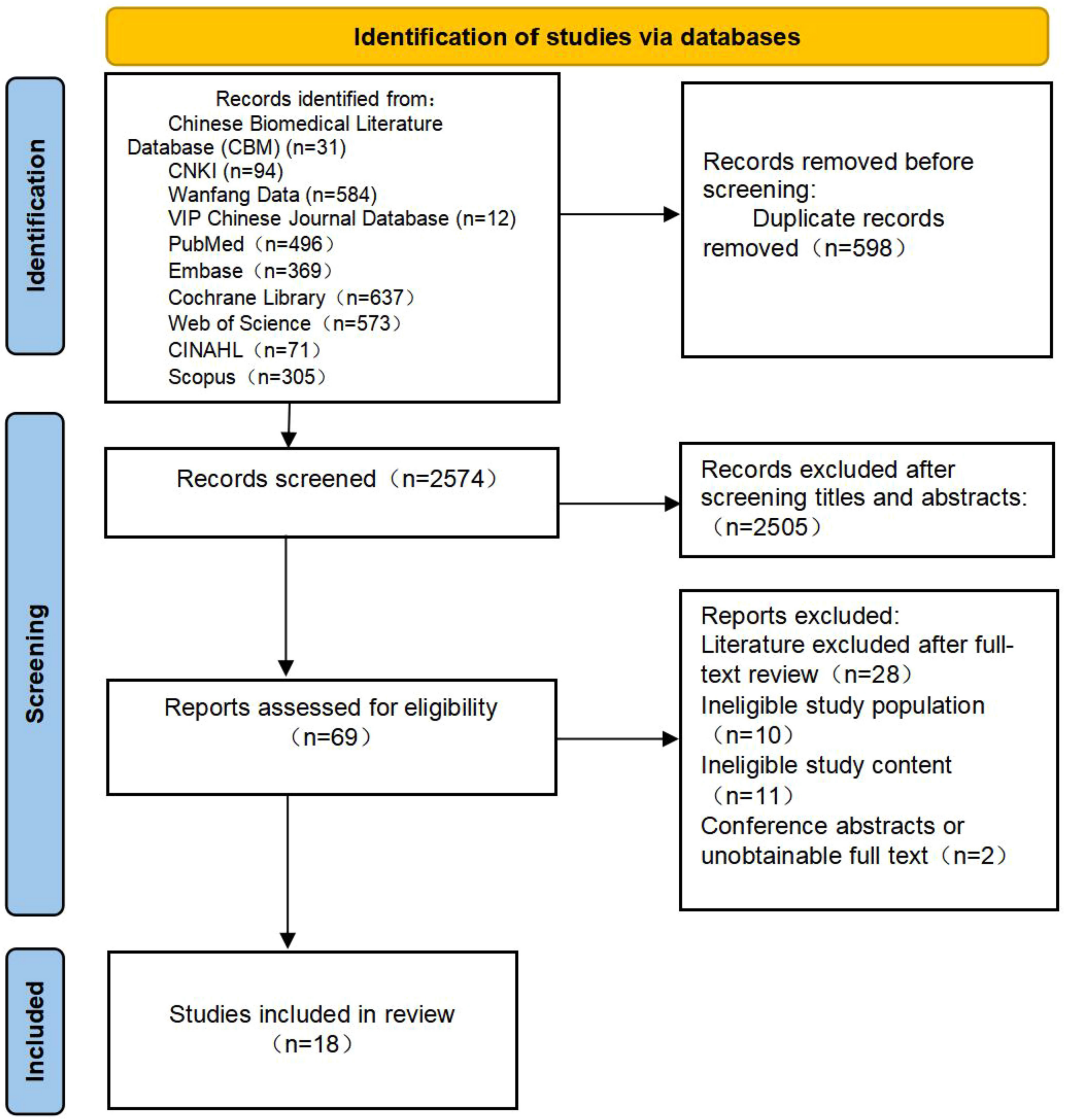
PRISMA flow diagram.

A total of 3172 records were identified through database searching. After removing duplicates, 2574 records remained. Following title and abstract screening, 2,505 records were excluded. Sixty-nine full-text articles were assessed for eligibility, and 51 were excluded. The main reasons for exclusion were non-qualitative study designs (*n* = 28), inappropriate study populations (*n* = 10), irrelevance to generative AI (*n* = 11), and unavailable full texts (*n* = 2). For the two studies with unavailable full texts, the authors were contacted and multiple institutional databases were searched; however, the articles could not be obtained. Ultimately, 18 studies were included in the meta-synthesis (13–15, 21–35). The literature screening process is presented in Figure 1. Of the included studies, 3 were in Chinese (22, 23, 31) and 15 were in English (13–15, 21, 24–30, 32–35), with 3 of them being mixed-methods studies (31–33), 2 being phenomenological studies (14, 24), and 13 being descriptive qualitative studies (13, 15, 21–23, 25–30, 34, 35).

### 3.2 Basic characteristics of the included studies

The basic characteristics of the included studies are presented in Table 1.

**TABLE 1:** Basic characteristics of the included studies.

Studies	Country	Study design	Data collection methods	Participants	Phenomenon of interest	Main findings
Ma et al. (23)	China	Descriptive qualitative study	Semi-structured interviews	15 nursing undergraduate students	To explore nursing students’ perceptions and experiences of using large language model chatbots in nursing education scenarios	Diverse user experiences; differentiated Usage scenarios; Potential challenges in use; Self-Perceived needs and expectations
Liu et al. (22)	China	Descriptive qualitative study	Semi-structured interviews	14 nursing postgraduate students	To understand the real experience of nursing postgraduates using artificial intelligence-generated content (AIGC) tools in scientific research	Strong interest in AIGC tools; perceived benefits from AIGC tool use; concerns regarding AIGC tool utilization
Lam et al. (30)	Canada	Descriptive qualitative study	Focus group interviews	16 nursing undergraduate students	To explore nursing students’ views on the use of ChatGPT in academics	Use as a supportive tool; risk of loss of basic skills and competencies; risks to professional reputation and academic integrity; need for further education and resources
Yuan et al. (14)	China	Phenomenological study	Semi-structured interviews	36 nursing undergraduate students	To understand nursing undergraduates’ perceptions of the advantages, disadvantages, opportunities and threats of generative artificial intelligence in learning activities	Strengths; weaknesses; Opportunities; threats
Gümüş and Dolu (15)	Turkey	Descriptive qualitative study	Semi-structured interviews	24 nursing undergraduate students	To explore undergraduate nursing students’ responses to an integrated ChatGPT transcultural nursing course and its impact on the learning process	Opportunities in curriculum design; cultural sensitivity; the role of ChatGPT
Shen et al. (29)	China	Descriptive qualitative study	Semi-structured interviews	17 undergraduate nursing students	To explore nursing students’ experiences and perceptions of using GenAI tools in developing nursing care plans	Experiences of using GenAI; efficiency in nursing case analysis; prompt design and its role; privacy and data security; emotional understanding toward GenAI; changes in learning approaches and professional identity
Han et al. (28)	US	Descriptive qualitative study	online questionnaire with semi-structured open-ended questions	99 undergraduate nursing students	To understand nursing students’ unique perceptions of GenAI in nursing education	Reasons for use; perceived advantages of GenAI and assistive AI in nursing education; disadvantages; perceived support needs for the use of GenAI and assistive AI in nursing education
Summers et al. (27)	Australia	Descriptive qualitative study	Semi-structured interviews	13 undergraduate nursing students	To explore nursing students’ perspectives on the application and potential integration of generative AI tools in nursing education	Educational impacts of AI tools; equitable learning environments; ethical considerations; technology integration; safety and practicality; generational differences
Ma et al. (26)	China	Descriptive qualitative study	Semi-structured interviews	24 undergraduate nursing students	To explore nursing students’ perceptions and experiences of using large language models (LLMs) in nursing study	Attitudes toward large language models; subjective norms; perceived behavioral control
Shen et al. (25)	China	Descriptive qualitative study	Semi-structured interviews	16 undergraduate nursing students	To explore undergraduate nursing students’ experiences and acceptance of NurCaseAI in clinical reasoning	Perceived system capabilities and limitations of NurCaseAI; learning enhancement benefits; challenges in interaction efficiency; limited emotional engagement; strong system acceptance
Siah et al. (21)	2Singapore	Descriptive qualitative study	Semi-structured focus group interviews	14 undergraduate nursing students	To understand students’ experiences regarding academic integrity and authenticity of work in relation to GenAI	Integration with learning; trust and credibility of GenAI information; ethical considerations; addressing academic integrity issues; guidance on GenAI use
Aslan et al. (24)	Turkey	Descriptive phenomenological study	Semi-structured online interviews	13 postgraduate nursing students	To explore postgraduate nursing students’ experiences with ChatGPT in nursing education	Various opportunities related to ChatGPT; hesitations; suggestions for the future
Wu et al. (31)	China	Mixed-methods study	One-on-one semi-structured interviews	8 vocational nursing students	To investigate the current use of generative AI among vocational nursing students, as well as its positive impacts and potential problems on their learning	Provides efficient and convenient access to knowledge; serves as a powerful learning assistant; concerns during generative AI use; expectations for in-depth application of generative AI
Uysal et al. (33)	Turkey	Mixed-methods study	Focus group interviews	23 undergraduate nursing students	To examine the impact of ChatGPT application in nursing education on the learning process	Importance of educational and teaching environments; limitations of AI-supported education; supportive role of AI and synergy of blended learning models
Abou et al. (32)	Saudi Arabia	Mixed-methods study	Semi-structured interviews	20 nursing students	To explore nursing students’ knowledge, perceptions, attitudes and concerns regarding ChatGPT	Use of ChatGPT; benefits and positive impacts of ChatGPT in academia; weaknesses and concerns about ChatGPT in academia; suggestions for future improvement of ChatGPT in academic settings
Jiang et al. (13)	China	Descriptive qualitative study	Structured interviews	16 postgraduate nursing students	To explore nursing postgraduate students’ experiences and perceptions of using generative artificial intelligence tools.	Performance expectancy; effort expectancy; social influence; usage attitudes and behaviors; boundaries to generative artificial intelligence adoption
Stratton-Maher and Kelly (34)	Australia	Descriptive qualitative study	Focus group interviews	48 first-year nursing students	To explore how first-year nursing students engaged with ChatGPT during an assessment task, and to understand the experiences, challenges, and perceptions they reported regarding its use in academic work.	initial confusion; developing skills; evaluating reliability; negotiating academic integrity; recognizing future benefits; valuing support
Huang et al. (35)	China	Descriptive qualitative study	Semi-structured interviews	20 undergraduate nursing students	To explore the experiences of nursing students in Taiwan who used ChatGPT during their courses	Convenient artificial intelligence (AI) assistant; Enhancing self-learning effectiveness; Challenges of ChatGPT usage;Gap between ChatGPT and nursing

### 3.3 Quality evaluation of the included studies

Seven studies were rated as A, and the remaining 11 studies were rated as B. In 11 studies (14, 15, 21, 23, 25, 27–29, 31, 33, 35), it was unclear whether the researchers adequately described their own backgrounds and values. In 4 studies (21, 27, 33, 35), the influence of the researchers on the research process, as well as the impact of the research on the researchers, was not clearly reported. The quality evaluation of the included studies is presented in Table 2.

**TABLE 2:** Methodological quality assessment of the included studies.

Included Studies	➀	➁	➂	➃	➄	➅	➆	➇	➈	❿	Quality rating
Ma et al. (23)	Yes	Yes	Yes	Yes	Yes	NO	Yes	Yes	Yes	Yes	B
Liu et al. (22)	Yes	Yes	Yes	Yes	Yes	Yes	Yes	Yes	Yes	Yes	A
Lam et al. (30)	Yes	Yes	Yes	Yes	Yes	Yes	Yes	Yes	Yes	Yes	A
Yuan et al. (14)	Yes	Yes	Yes	Yes	Yes	NO	Yes	Yes	Yes	Yes	B
Gümüş and Dolu (15)	Yes	Yes	Yes	Yes	Yes	NO	Yes	Yes	Yes	Yes	B
Shen et al. (29)	Yes	Yes	Yes	Yes	Yes	NO	Yes	Yes	Yes	Yes	B
Han et al. (28)	Yes	Yes	Yes	Yes	Yes	NO	Yes	Yes	Yes	Yes	B
Summers et al. (27)	Yes	Yes	Yes	Yes	Yes	NO	NO	Yes	Yes	Yes	B
Ma et al. (26)	Yes	Yes	Yes	Yes	Yes	Yes	Yes	Yes	Yes	Yes	A
Shen et al. (25)	Yes	Yes	Yes	Yes	Yes	NO	Yes	Yes	Yes	Yes	B
Siah et al. (21)	Yes	Yes	Yes	Yes	Yes	NO	NO	Yes	Yes	Yes	B
Aslan et al. (24)	Yes	Yes	Yes	Yes	Yes	Yes	Yes	Yes	Yes	Yes	A
Wu et al. (31)	Yes	Yes	Yes	Yes	Yes	NO	Yes	Yes	Yes	Yes	B
Uysal et al. (33)	Yes	Yes	Yes	Yes	Yes	NO	NO	Yes	Yes	Yes	B
Abou et al. (32)	Yes	Yes	Yes	Yes	Yes	Yes	Yes	Yes	Yes	Yes	A
Jiang et al. (13)	Yes	Yes	Yes	Yes	Yes	Yes	Yes	Yes	Yes	Yes	A
Stratton-Maher and Kelly (34)	Yes	Yes	Yes	Yes	Yes	Yes	Yes	Yes	Yes	Yes	A
Huang et al. (35)	Yes	Yes	Yes	Yes	Yes	NO	NO	Yes	Yes	Yes	B

➀ Is the philosophical foundation and methodology consistent? Yes/No; ➁ Is the methodology aligned with the research problem or research objective? Yes/No; ➂ Is the methodology consistent with the data collection method? Yes/No; ➃ Is the methodology consistent with the representativeness of the data and the data analysis method? Yes/No; ➄ Is the methodology consistent with the interpretation of the results? Yes/No; ➅ Does the study explain the researchers’ own background from the cultural context and values? Yes/No; ➆ Does the study explain the influence of the researcher on the study or the impact of the study on the researcher? Yes/No; ➇ Are the research subjects typical? Yes/No Does the study sufficiently reflect the research subjects and their viewpoints? Yes/No; ➈ Has the research been approved by the relevant ethics review board? Yes/No; ❿ Are the conclusions derived from the analysis and interpretation of the data? Yes/No.

### 3.4 Meta-synthesis results

Through iterative reading and analysis of the 18 included studies, 49 themes were identified and grouped into 13 categories. These categories were further synthesized into four integrated findings. The synthesis process and the development of themes, categories, and integrated findings are presented in Table 3.

**TABLE 3:** Research findings integration process.

Themes	Categorie	Integrated findings
Result 1: Used as a support tool (13, 28, 30, 34, 35)	Category 1: Positive experiences of efficiency enhancement and cognitive expansion	Integrated result 1: Dual experience of empowerment and challenges
Result 2: Expands knowledge horizons (14, 21, 22)		
Result 3: Helps students view problems from multiple perspectives (25, 31, 35)		
Result 4: Provides instant feedback (24, 28, 29)		
Result 5: Creates an inclusive interactive environment (25, 26)		
Result 6: Possesses data processing and knowledge integration capabilities (13, 14, 29, 30)		
Result 7: Improves efficiency and productivity (24, 26, 29, 34, 35)		
Result 8: Simplifies complex concepts (14, 26)		
Result 9: Trains communication skills (22, 23, 25, 35)		
Result 10: Low-quality and inaccurate outputs (13, 14, 28, 34, 35)	Category 2: The burden of reliability doubts and validation	
Result 11: Imposes a verification burden (13, 24, 33, 35)		
Result 12: Blurs the boundaries of academic integrity (15, 27, 30)	Category 3: Ethical anxiety arising from blurred boundaries of academic integrity	
Result 13: Improper use endangers reputation and academic integrity (23, 27)		
Result 14: Increases the difficulty of AI discrimination (27, 30)		
Result 15: Foster a mentality of taking chances (13–15, 35)		
Result 16: Loss of basic skills and abilities (29, 32)	Category 4: Concerns about skill dependency and skill degradation	
Result 17: Degradation of core competencies (21, 30, 31)		
Result 18: Who is learning? Me or AI? (24)		
Result 19: Limited emotional engagement (14, 15, 30)	Category 5: Tool-based interactions lacking emotional understanding	Integrated result 2: Internal conflict between technology and nursing humanism
Result 20: Limitations in emotional understanding (14, 26, 29, 35)		
Result 21: Cannot fully replace teachers (25, 32, 35)		
Result 22: Generated information lacks personalization (27, 29, 35)	Category 6: Exploring the boundaries of AI assistance and replacement in clinical decision-making	
Result 23: Triggers brainstorming (31)		
Result 24: Facilitates case analysis (25, 29, 31)		
Result 25: Insufficient digital literacy (14, 23, 26, 34)	Category 7: Loss of control due to insufficient digital literacy	Integrated result 3: User experience differentiation amid practical constraints
Result 26: Technical barriers (14, 28, 34)		
Result 27: High requirements for input prompts (25, 26, 28)		
Result 28: High usage threshold (13, 14, 25, 34)		
Result 29: Updates too rapidly (13, 14)		
Result 30: Subjective norms (13, 27, 32)	Category 8: Environmental impacts and non-standard ruless	
Result 31: Unclear legal liability and ethical boundaries (28, 30, 32)		
Result 32: Intergenerational differences (23, 27)		
Result 33:Inevitable trend (13)		
Result 34: Digital divide (14, 22, 30)	Category 9: Accessibility inequality and privacy concerns as limiting factors	
Result 35: Equity gaps (14, 29)		
Result 36: Privacy and data security (13, 28, 31)		
Result 37: Requires further education and resources (13, 14, 23, 29, 34)	Category 10: Urgent demand for structured training and clear guidelines	Integrated result 4: General demand for supporting systems and educational reform
Result 38: Establish standardized usage guidelines (14, 30)		
Result 39: Improve AI usability (14, 23, 29)	Category 11: Industry expectation for the development of nursing-specific AI tools	
Result 40: Develop AI tools tailored for medical and nursing care (14, 25, 35)		
Result 41: Interdisciplinary integration and collaboration (21, 32)		
Result 42: Establish clear moral and ethical guidelines (25, 33)	Category 12: Shared call for ethical frameworks and policy norms	
Result 43: Provide legal safeguards (21, 24, 34)		
Result 44: Provide policy norms (24, 33)		
Result 45: Plagiarism detection (24, 34)		
Result 46: Enhance academic integrity reminders (25, 33)		
Result 47: Hybrid learning model (25, 33)	Category 13: Endorsement of a collaborative hybrid learning ecosystem	
Result 48: Enhances classroom engagement and fun (25, 33)		
Result 49: Teachers use AI to assist teaching (25, 33)		

#### 3.4.1 Category 1: positive experiences of efficiency enhancement and cognitive expansion

Nursing students generally believed that generative AI tools significantly improved learning, research, and work efficiency. As writing and academic support tools, they assisted students in generating outlines, revising grammar, refining language, and enhancing the quality and structure of academic writing [“I think like it can help in terms of writing assistance, but it can help in writing more academic papers or general outlines or refining like the language chosen to make arguments a bit stronger…. making paper look more professional.”(30)]. As personalized learning partners and “virtual” research assistants, generative AI tools provided a non-judgmental and readily accessible environment for asking questions, generating ideas, supporting revision, and expanding knowledge horizons [“The textbook is limited, but AI lets me explore more extracurricular knowledge—things you do not get in class.”(14)]. Generative AI also functioned as an effective information-processing tool. It rapidly summarized complex topics, organized fragmented knowledge, and provided multiple perspectives on a subject, thereby reducing the time required for information retrieval and synthesis [“It helps me quickly check the paper for grammatical errors, allowing me to spend more time on writing. “ (26)]. Students particularly appreciated the ability of generative AI to organize fragmented information and generate preliminary analytical frameworks. This capability provided a clear starting point and structured guidance for complex nursing case analyses and comprehensive assignments [“It was really helpful how GenAI could instantly generate an analytical framework. I felt like I had a basic blueprint to work from, which saved me so much time refining my ideas.”(29)]. For postgraduate students, generative AI served as a temporary source of academic support when access to supervisors was limited. It provided immediate guidance on research ideas and literature organization [“As you know, accessing an educator is difficult at certain times at the postgraduate level. This issue may arise from both sides, the students and the educators. While ChatGPT is not a real supervisor, it allowed me to ask questions and also provided guidance on certain challenging points, especially in developing my research question.”(24)].

#### 3.4.2 Category 2: the burden of reliability doubts and validation

While students acknowledge the efficiency of generative AI, they express ongoing concerns about the accuracy, timeliness, comprehensiveness, and reliability of its generated content. Students recognize the need to adopt a cautious approach to AI-generated information and avoid accepting it uncritically [“needs to be taken with a grain of salt… they are good in certain context, but you cannot blindly accept it.”(27)]. Compared with the high level of trust placed in teachers, students place less trust in the outputs generated by AI. As a result, they often spend additional time and effort verifying information [“We need to verify information from Chat-GPT all the time. This makes it difficult for us. But when the teacher tells us, we accept it as true.”(33)], Some students also reported feelings of uncertainty, discomfort, and anxiety when using AI-generated content [“Yes, ChatGPT was a helpful tool for us, although verification of the content provided by this tool has to be verified every time for accuracy. This uncertainty makes me feel awkward. I am always unsure of the accuracy of the content, and there are also ethical considerations.”(24)], the need for continuous verification increased their cognitive burden during the learning process.

#### 3.4.3 Category 3: ethical anxiety arising from blurred boundaries of academic integrity

The use of generative AI has raised significant ethical concerns and uncertainty regarding academic integrity among nursing students. The primary dilemma involves defining the boundary between appropriate use and academic misconduct. For example, students continue to debate whether using generative AI for idea generation, text revision, or assignment completion constitutes plagiarism. Most students considered the use of generative AI acceptable when the core ideas and thinking processes originated from the learner, such as for grammar correction or language refinement. However, directly copying AI-generated content or relying on it to complete assignments or examinations was generally regarded as inappropriate [“When you ask a question, let’s say for written assignment, most people (would) just put it into ChatGPT, and look at the answer given, and see where they can or how they can use the outline given to start their research. To me, I don’t have an issue with that. It’s about how you use information in the ethical way to get the information they want. So what’s not right is, you copy and paste the whole thing like into your assignment.”(21)]. As AI-generated outputs become increasingly sophisticated, students perceived growing difficulties in distinguishing between human- and AI-generated work. This further intensified concerns about the future of academic integrity [“But I think like as time goes on and chat GPT develops more, it’ll become like more difficult to detect whether or not something was written by an individual or by artificial intelligence.”(30)]. Some students acknowledged that the convenience of generative AI could encourage shortcut-taking behaviors, potentially reducing motivation to complete academic tasks independently. A few even described it as a “cheating tool.” [“There are good and bad aspects. For example, I thought, ‘Well, ChatGPT will handle it; I don’t need to work, it can do it for me.”(15)]. Despite concerns that peers might gain unfair academic advantages through the use of generative AI, many students emphasized the importance of personal integrity and long-term learning outcomes over short-term grade comparisons [“I don’t think it (grades) matters too much, because, in the first place, I don’t really like compare grades. At the end of the day for me, the fact that I do my own work. That’s more than enough for me.”(21)].

#### 3.4.4 Category 4: concerns about skill dependency and skill degradation

Students were generally aware of the potential long-term risks of overreliance on generative AI for skill development. They expressed concerns that excessive dependence on AI for writing, information integration, case analysis, and research-related thinking could weaken critical thinking, independent analysis, deep learning, creativity, and problem-solving abilities [“It feels like my ability to think independently has declined because it is so fast and comprehensive. As a result, I become very dependent on it to help me get a better assignment or presentation. “(31)]. This dependence was described as “bypassing the learning process,” which may lead students to do only the “bare minimum to obtain a degree,” fostering “lazy learners” and limiting opportunities for alternative learning pathways. The convenience of rapid information retrieval also led some students to feel that they were “not truly putting in the effort to learn,” prompting them to question whether knowledge was being genuinely internalized [“At first, I felt that everything was very good. Yes, I still believe that using ChatGPT offers opportunities. However, after using it for 3 to 4 days, I began to question whether my knowledge was truly expanding. This is a critical question we must ask ourselves to ensure we are using this tool effectively.”(24)].

#### 3.4.5 Category 5: tool-based interactions lacking emotional understanding

Students clearly recognized the limitations of generative AI in providing emotional interaction within nursing learning and practice contexts. They noted that although AI is efficient, it remains a task-oriented tool. The emotional support and care-related advice generated by AI was often perceived as vague, superficial, and insufficiently personalized, making it difficult to address complex human emotions and specific situational needs. Generative AI was also viewed as unable to replicate the human connection established through trust, understanding, and non-verbal communication between nurses and patients [“It cannot provide emotional support to patients. It is merely a cold tool and can’t put itself in the patient’s shoes.”(26)]. In simulated learning environments, students reported that generative AI often failed to recognize and respond appropriately to emotions such as frustration and confusion. As a result, interactions were perceived as mechanical and lacking empathy [“When I typed “this question is too hard,” the system didn’t recognize my frustration and just repeated the question. It felt like arguing with a machine” (25)]. By contrast, students emphasized that teachers’ body language, facial expressions, tone of voice, and personal experiences played an important role in promoting understanding, memory, and emotional engagement. These human elements were considered irreplaceable by current generative AI technologies [“I need to communicate with the teacher’s gestures and facial expressions, for example.”(33)].

#### 3.4.6 Category 6: exploring the boundaries of AI assistance and replacement in clinical decision-making

In clinical learning and practice contexts, students actively explored the appropriate role of generative AI. They generally viewed AI as a valuable assistant for providing informational references, generating preliminary frameworks, and identifying overlooked details. In nursing case analyses, generative AI was perceived as helpful in offering structured analytical pathways and targeted feedback, serving as a “guide” or “another pair of eyes.” [“The case presentation was concise and smooth. Each section connected logically to the next, which definitely helped me grasp the concepts better.”(25)]. In group learning activities, differences in outputs generated by various AI tools often stimulated discussion and critical thinking among students [“In class, the teacher will assign case discussion tasks. The Gen AI tools used by the group members will be different, and sometimes the answers they analyze are not the same. At this time, we will combine the case and the knowledge from the textbook to discuss who is right and who is wrong, or which answer is more reasonable.”(31)]. However, students also recognized the limitations of generative AI in clinical decision-making. AI-generated content often lacked the nuanced judgment, individualized adjustments, and patient-centered considerations required in clinical practice. As a result, substantial revision and adaptation were often necessary to ensure that recommendations aligned with patients’ specific needs and circumstances [“Generated plans look complete at a glance, but when you dig deeper, there’s a shortage of individualized information. We had to revise and tailor the content significantly to match our patient’s exact condition.”(29)].

#### 3.4.7 Category 7: loss of control due to insufficient digital literacy

Students experienced frustration and a reduced sense of control when using generative AI because of limited digital literacy. Some were unfamiliar with the functions, operating principles, and terminology of AI tools [“The long text it generates has a strange style and inconsistent formatting. I end up rewriting everything myself.”(14)]. Others reported a limited understanding of how generative AI works and of its potential applications [Actually, I don’t really understand its operating principle, nor do I know many of its potential functions (23)]. Students particularly struggled with prompt formulation and found it difficult to ask effective questions that would generate relevant and high-quality responses [I’ve heard that using prompt words can make things much easier, but I don’t know how to apply them (31)]. Frequent system updates and complex interfaces also posed challenges for some users [“Updating an AI system means downloading several gigabytes—and I cannot use it while it updates. Then I have to relearn how to use it.”(14)]. Variations in digital literacy may further widen disparities in students’ ability to benefit from generative AI.

#### 3.4.8 Category 8: environmental impacts and non-standard rules

Students’ use of generative AI was shaped by a complex interplay of institutional, social, and individual factors. On the one hand, many students reported uncertainty due to the lack of clear institutional policies, usage guidelines, and ethical standards. As a result, they were often unsure about which uses of AI were acceptable or encouraged. [“It’s important to know the limits—when should we use ChatGPT, and when is it better to go with traditional methods?”(32)]. On the other hand, social influences played an important role. Media exposure and the adoption of AI by peers, senior students, and experts increased students’ curiosity and motivation to use these tools, sometimes creating a fear of missing out (FOMO) [“Many excellent students or role models around me are using ChatGPT and Bing-Chat, which has greatly increased their productivity, and if I don’t use them, I feel like I might fall behind.”(26)]. Conversely, institutional restrictions, ambiguous policies, and concerns about potential penalties created additional pressure. These factors sometimes led students to use AI secretly, feel confused, or limit their exploration of AI applications [“It can be fatally damaging if a reviewer finds out that I used LLMs to ghostwrite papers, however, I must admit that I sometimes use them secretly.”(26)]. Individual interests, learning needs, and generational differences also influenced patterns of AI use and levels of acceptance [“I’m quite fond of these things. I learned about ChatGPT through Douyin. When some tech bloggers mentioned that such a tool had emerged, I thought it was very amazing at that time and started using it.”(22)]. In addition, many students viewed generative AI as an inevitable development in the digital era. They believed that learning to use AI effectively was necessary to remain competitive in future academic and professional environments [“You must adapt, or you’ll be left behind.”(13)].

#### 3.4.9 Category 9: accessibility inequality and privacy concerns as limiting factors

The adoption of generative AI was constrained by concerns related to both accessibility and data security. A notable digital divide existed, as students faced unequal access to advanced AI tools because of financial constraints, geographic restrictions, and differences in institutional resources [“Many public media accounts are currently promoting the considerable potential of AI in research and writing; however, I use it relatively little at present, mainly due to cost concerns.”(22)], geographic access restrictions, and differences in resources between institutions [“Our university does not even have an AI lab. Meanwhile, top schools use AI to simulate clinical scenarios—we have not even had a basic coding class.”(14)]. Concerns about privacy and data security were also widespread. Students worried that sensitive patient information, personal learning materials, or unpublished research data might be stored, misused, or disclosed by AI platforms [“I always wonder if all these cases we’re typing in are somehow being stored by the AI. Like, where does all that information actually go?”(29)]. While some students had limited awareness of data protection risks, others were highly cautious and chose to avoid certain functions or restrict their use of AI because of privacy concerns [“If I upload my data to it today for training, it might pass that data on to others tomorrow—I’m not sure whether that could happen. Therefore, I prefer not to use it for data processing.”(22)].

#### 3.4.10 Category 10: urgent demand for structured training and clear guidelines

Students consistently expressed a strong need for structured education and training related to generative AI. They hoped that educational institutions and instructors would provide clear guidance on the ethical boundaries of AI use, effective application strategies, potential risks, and the identification of reliable information sources [“AI enabled tools are,… here now, they’re accessible now and they’re going to be accessible for the newer generations going throughout the school…. It seems like it would require just some education about… ethics, rules, boundaries,… when to use it, when not to use it, what’s right, what’s wrong.”(30)]. Students also expected such training to be introduced early in their education through courses, workshops, and lectures. They emphasized the importance of integrating AI-related content into nursing curricula [“I hope our school can offer more courses that combine AI with nursing. It would help us expand our thinking and learn crossdisciplinary knowledge.”(14)]. Students believed that this support would enable them to use generative AI more responsibly, make informed decisions, and benefit more effectively from AI-assisted learning.

#### 3.4.11 Category 11: industry expectation for the development of nursing-specific AI tools

Students expressed strong expectations for the development of more advanced and reliable nursing-specific AI tools. They hoped for AI systems built on authoritative and up-to-date nursing knowledge bases, including clinical guidelines, case repositories, and research evidence, to improve the reliability, relevance, and practical value of AI-generated content [“I hope there will be an AI knowledge base specifically designed for nursing, continuously updating nursing cases, research reports, and related data—authoritative and comprehensive.”(14)]. Students particularly valued AI tools that were closely aligned with nursing curricula and capable of providing accurate support for clinical reasoning. They also anticipated further improvements, including the integration of multimedia resources, enhanced interactivity, and greater accuracy in image generation [“Current tools such as ChatGPT-4 and ERNIE Bot can generate images of anatomical structures and clinical scenarios based on prompts; however, their accuracy remains insufficient, and I hope this aspect can be further improved.”(23)]. In addition, students expressed interest in the future expansion of innovative AI applications within nursing education and practice [“In my future nursing practice, I believe there will be a strong need for innovative and newly developed functionalities.”(31)].

#### 3.4.12 Category 12: shared call for ethical frameworks and policy norms

Faced with the ethical and legal challenges associated with generative AI, students recognized the need for regulatory frameworks at the policy, institutional, and professional levels. They called for clear ethical guidelines, legal safeguards, and governance policies addressing issues such as data privacy, clinical accountability, academic integrity, and algorithmic bias. These measures were viewed as essential for the safe and responsible use of AI [“Legal safeguards are equally important. I believe that as technology advances and legal regulations improve, we can strike a balance where AI’s advantages are fully utilized while ensuring patient privacy and data security.”(29)]. Students also emphasized the importance of educator oversight, guidance, and experience sharing, particularly in the absence of clear standards. Many hoped that educators and supervisors would help review and validate AI-generated content, especially at the postgraduate level [“Yes, I had access to greater information and resources than I had expected, although it felt like something was missing. I needed someone to verify the content I developed using ChatGPT. It would be beneficial if the educators or supervisors reviewed the content at regular intervals as there was so much information and I had difficulty organizing it.”(24)].

#### 3.4.13 Category 13: endorsement of a collaborative hybrid learning ecosystem

Students generally agreed that generative AI should complement rather than replace conventional teaching. They emphasized the importance of preserving the core value of face-to-face education, particularly its role in fostering social interaction, non-verbal communication, emotional connection, and a structured learning environment [“First, we can take the lesson from our educator and then we can complete the information we are curious about or the information that we could not get in the lesson with artificial intelligence.”(33)]. At the same time, students expected generative AI to support pre-class preparation, post-class review, personalized learning, and access to additional resources. They envisioned a collaborative learning model characterized by “teacher-led, AI-assisted, and student-centered” education. This hybrid approach was perceived as enhancing learning efficiency and engagement while preserving the humanistic values of education and promoting deeper learning experiences [“If a course relies solely on content summarized by the instructor, it may feel somewhat monotonous and less engaging for students. However, incorporating elements of artificial intelligence can enhance our motivation to learn. For example, in an innovation and entrepreneurship course, the instructor asked us to use tools like Doubao and Jianying to create scenario-based videos. I felt like a director, which deepened my impression and understanding of the course content.”(31)].

## 4 Discussion

### 4.1 Nursing students’ experience with generative AI combines empowerment and challenges

This study shows that, nursing students generally experience both “efficiency reliance” and “risk anxiety” when using GenAI during learning. While they benefit from its significant enhancement of information processing and writing assistance, they are also concerned about information reliability, blurred boundaries of academic integrity, and the potential decline in critical thinking abilities. Notably, these benefits and concerns are closely interconnected. The same characteristics that make GenAI attractive—such as rapid information retrieval and immediate feedback—may also contribute to overreliance and reduced cognitive engagement. This tension arises from the inherent limitations of GenAI technology, including “hallucinations” and limitations in information currency, the lag of educational ethics behind technological development, and the conflict between the tool’s convenience and the active thinking required for deep learning. Therefore, educators must effectively manage and promote students’ use of this technology (26). First, nursing education institutions should quickly formulate and issue specific academic integrity guidelines for GenAI use, clearly presenting acceptable usage and academic misconduct through case studies (2, 14, 21, 23, 28), to alleviate students’ confusion and anxiety at the institutional level. Nursing instructors can also adopt alternative assessment strategies, such as oral evaluations, reflective writing, or collaborative assignments, to reduce dependence on AI outputs and encourage deeper student engagement (24). Finally, it is essential to clearly communicate in education that “GenAI is a “partner” or “scaffold” to assist thinking, not a “crutch” to replace independent thinking,” encouraging students to use tool-generated outputs as starting points for deepening discussions and inspiring ideas, rather than endpoints for thinking.

### 4.2 The use of generative AI creates inherent tensions between technology application and nursing humanism among nursing students

Generative AI in nursing education presents a fundamental contradiction: its powerful information-processing capacity contrasts sharply with the emotional connection and individualized care central to nursing practice, creating an insurmountable gap (29). Notably, students do not simply reject AI because of this limitation; rather, they appear to view AI as useful for supporting technical and cognitive tasks while recognizing that humanistic aspects of nursing remain irreplaceable. This finding suggests that nursing students are actively negotiating the boundaries between technological efficiency and human-centered care. The technical nature of generative AI is based on pattern-based processing of massive amounts of data, lacking genuine “understanding” of human emotions, specific contexts, and ethical nuances (25). In contrast, nursing practice is inherently human-centered, uncertain, and an art form, which leads to a logical conflict between the two. Therefore, nursing educators must recognize that generative AI cannot replace the social and emotional dimensions of learning derived from interpersonal interactions and teacher-student relationships, and help nursing students achieve a relative balance (28). The focus should be on training students to use AI to quickly access information and generate initial analyses, but they must personalize, integrate emotions, and ultimately make clinical judgments. It is also important to encourage the development or introduction of AI simulation systems tailored to nursing that incorporate rich case scenarios (29, 30), thereby enhancing learning immersion (36), while guiding students to critically compare AI-generated responses with authentic caregiving experiences. Furthermore, course design and evaluation should continue to emphasize communication skills, empathy, and ethical decision-making.

### 4.3 The acceptance of generative AI by nursing students is significantly influenced by external contexts and individual differences

Nursing students’ acceptance and use of generative AI are not homogeneous but are shaped by differences in digital literacy, environmental norms, and resource accessibility. Students with limited digital skills often experience frustration when using AI tools (14, 15, 24); while policy ambiguity, ethical uncertainty, and unequal access resulting from the digital divide further influence adoption (14, 28). Concerns about data privacy and information security also restrict the depth and scope of AI use (29). These findings suggest that students’ engagement with generative AI is influenced not only by the technology itself but also by the broader educational and social context in which it is used.

Notably, although students reported multiple barriers to AI use, they generally regarded generative AI as an inevitable component of future healthcare and nursing education. This finding reflects a pragmatic attitude toward technological change. Students appeared willing to engage with AI despite existing concerns because they perceived AI literacy as increasingly important for academic success and future professional development (13). Meanwhile, nursing is a profession with high ethical sensitivity and rigorous clinical standards. It is essential to formulate and implement sound ethical guidelines to address students’ prevalent concerns over data privacy and information security. Otherwise, the improper use of Gen AI will directly undermine three core pillars of nursing: professional accountability, evidence-based practice (EBP) integrity, and clinical decision-making safety. In future clinical practice, overreliance on GenAI when developing nursing plans may weaken students’ critical thinking and professional judgment, increase risks in clinical decision-making, and undermine professional accountability and ethical practice.

Based on these findings, nursing schools should provide structured education on generative AI through dedicated courses or workshops covering fundamental AI concepts, prompt engineering, critical appraisal, data security, and professional ethics, thereby enhancing digital literacy and reducing skill disparities among students. Institutions should also improve access to appropriate AI tools, particularly in resource-limited settings, by reducing technical barriers and promoting cross-regional collaboration (15), Given that equitable access to educational resources is essential for the widespread adoption of AI in education (37), schools should establish clear guidelines for the safe use of AI, including the protection of patient privacy and responsible data handling (36). In addition, educators should adopt differentiated teaching strategies that address students’ varying levels of digital competence, technological anxiety, and ethical concerns, while integrating professional values, accountability, and evidence-based practice throughout AI-related education. Such efforts may help students develop responsible, critical, and professional AI-use habits and better prepare them for the continued advancement of smart healthcare.

### 4.4 Nursing students expect the development of a humanism-centered, technology-supported hybrid AI education ecosystem

Research indicates that nursing students are not passive recipients of technology but hold forward-looking and constructive expectations regarding the integration of generative AI into nursing education. They express a strong need for structured training, to enhance AI-related competencies and ethical awareness, expect the development of nursing-specific tools to increase professional applicability (35), and call for the establishment of clear ethical and policy frameworks to ensure safety (14). Nursing students generally agree with the construction of a human-AI collaborative hybrid learning ecosystem. These expectations stem from their firsthand experience as users, recognizing both the potential and limitations of AI in practice. They expect educational institutions to guide the responsible use of technology in ways that support, rather than undermine, the humanistic values and deep learning central to nursing education. Accordingly, nursing programs should systematically incorporate AI-related content into curricula (22, 23), and support the development of AI-assisted platforms based on authoritative nursing knowledge and clinical scenarios, particularly for case-based learning and skills training. Educators should be encouraged to use AI to support lesson preparation, personalized learning activities, and resource development, while maintaining a strong emphasis on interaction, empathy, and higher-order thinking in the classroom (31). Such an approach may enable educators to become effective designers of AI-enhanced nursing education (29). By enhancing educators’ ability to integrate AI into teaching, clarifying their new roles, and providing solid institutional and resource support, the education system can effectively respond to students’ collective expectations for structured training, ethical guidance, and a hybrid learning ecosystem, thus systematically promoting the transformation of nursing education (21, 24).

### 4.5 Implications for tiered AI education across different nursing education levels

Existing evidence suggests that nursing students’ experiences with generative AI are shaped by their educational stage, learning needs, and professional development goals. While students across all educational levels recognize the potential of AI to improve learning efficiency, their expectations and patterns of use differ substantially. This finding indicates that a “one-size-fits-all” approach to AI education may be insufficient. Instead, the integration of generative AI into nursing education should be aligned with the developmental objectives of different educational levels, thereby supporting progressive competency development throughout the nursing education pathway.

For diploma-level nursing students, whose education primarily focuses on foundational knowledge and practical skill development (38), generative AI may serve as a supportive learning tool for clarifying concepts, organizing course content, and reinforcing basic clinical procedures through simulation and guided practice. In contrast, undergraduate nursing education places greater emphasis on integrating knowledge, analyzing clinical problems, and developing clinical reasoning skills (39). At this stage, AI can support case-based learning, knowledge synthesis, and clinical scenario analysis. However, students should be encouraged to maintain independent thinking and avoid overreliance on AI-generated outputs when addressing clinical questions. For graduate nursing students, whose training emphasizes scientific inquiry, innovation, and advanced professional development (40). Generative AI may function as a research support tool for literature exploration, idea generation, and academic writing. Nevertheless, the principles of independent thinking, originality, and academic integrity must remain central.

Taken together, these findings support the development of a tiered framework for AI integration in nursing education, with diploma programs emphasizing foundational skills, undergraduate programs focusing on clinical reasoning, and graduate programs prioritizing research and innovation. Future empirical studies are needed to evaluate the effectiveness of this stratified approach and to further refine educational guidelines for AI use across different stages of nursing education.

This study has several limitations. First, only Chinese and English literature was included, which may have introduced language bias. Second, studies with positive or “information-rich” findings are more likely to be published, potentially leading to publication bias. In addition, the included studies mainly focused on undergraduate and postgraduate nursing students, while research involving vocational nursing students was limited, which may affect the broader applicability of the findings. Furthermore, substantial differences existed among studies in terms of the generative artificial intelligence tools used, application scenarios, and educational contexts, which may have increased the heterogeneity of the results. Moreover, given the rapid development of generative artificial intelligence technologies, students’ experiences are highly time-sensitive, and the conclusions of this study should be updated in light of future emerging evidence.

## 5 Conclusion

This meta-synthesis explored nursing students’ experiences with generative AI-assisted learning and identified four integrated findings: dual experience of empowerment and challenges, internal conflict between technology and nursing humanism, user experience differentiation amid Practical Constraints, general demand for supporting systems and educational reform. The findings suggest that generative AI can enhance learning efficiency, academic support, and self-directed learning, while simultaneously raising concerns related to information reliability, academic integrity, overreliance on technology, and the erosion of humanistic values in nursing. These findings highlight the need to balance technological innovation with the core values of nursing education. Effective integration of generative AI requires not only the development of students’ AI literacy, critical thinking, and ethical awareness, but also the establishment of clear educational guidance, institutional policies, and context-sensitive support strategies across different educational levels. This study provides evidence to inform the responsible and educationally meaningful integration of generative AI into nursing education.

## Data Availability

Publicly available datasets were analyzed in this study. This data can be found here: https://pubmed.ncbi.nlm.nih.gov.
